# Clinically Suspected Tumefactive Demyelination Mimicking a Brain Tumor in a 10‐Year‐Old Boy: A Case Report

**DOI:** 10.1002/ccr3.73887

**Published:** 2026-11-06

**Authors:** Fatima Aman, Ahmad Ali, Muhammad Fawad Khan, Haq Nawaz Khan, Asim Shah, Ali Sher Erkin, Suleman Khan, Yasar Sattar, Aizaz Anwar Khalid

**Affiliations:** ^1^ Rehman Medical Institute Peshawar Pakistan; ^2^ Peshawar Medical College Peshawar Pakistan; ^3^ Hayatabad Medical Complex Peshawar Pakistan; ^4^ Khyber Medical College Peshawar Pakistan; ^5^ Kabul University of Medical Sciences (KUMS) Kabul Afghanistan; ^6^ Camden Clark Medical Center Parkersburg West Virginia USA; ^7^ West Virginia University Morgantown West Virginia USA

**Keywords:** brain tumor mimic, corticosteroid therapy, magnetic resonance imaging, open‐ring enhancement, pediatric demyelination, suspected tumefactive demyelination

## Abstract

After cranial surgery, a 10‐year‐old boy developed hand weakness, numbness, and fatigue. MRI showed frontoparietal lesions; biopsy lacked documented demyelination. Symptoms and lesions reportedly improved after methylprednisolone. Tumefactive demyelination was suspected, but discordant laterality, unavailable antibody testing, and brief follow‐up left the diagnosis unclassified.

## Key Clinical Message

Open‐ring enhancement and early regression after corticosteroids can support a provisional diagnosis of tumefactive demyelination in a child with tumor‐like brain lesions. Clinical localization, neuropathology, antibody testing, and longitudinal follow‐up are needed to refine the diagnosis; steroid responsiveness alone does not establish multiple sclerosis.

## 1 Introduction

Tumefactive demyelinating lesions are inflammatory central nervous system lesions, generally at least 2 cm in diameter, that can resemble brain tumors because of their size, edema, enhancement, and mass effect. Pediatric series demonstrate that these lesions can cause substantial diagnostic uncertainty and, in severe cases, intracranial hypertension requiring intervention [1, 2].

Tumefactive demyelination describes a lesion pattern rather than a diagnosis of multiple sclerosis (MS). It can occur with MS, myelin oligodendrocyte glycoprotein antibody‐associated disease (MOGAD), aquaporin‐4 antibody‐associated neuromyelitis optica spectrum disorder (NMOSD), or an otherwise unclassified inflammatory demyelinating event [3, 4]. Open‐ring enhancement, peripheral diffusion restriction, and relatively limited mass effect may favor demyelination over neoplasia, but no single conventional MRI feature is diagnostic [5].

Distinguishing a clinically suspected tumefactive demyelinating event from established MS is particularly important in children. Contemporary MS criteria require an appropriate clinical and paraclinical assessment and exclusion of better explanations; lesion size and steroid responsiveness alone are insufficient [6]. Incomplete antibody testing or nonspecific inflammatory histology should therefore be reflected in the diagnostic terminology.

We report a 10‐year‐old boy with clinically suspected tumefactive demyelination who presented after surgery for a presumed brain tumor. This case illustrates the difficulty of interpreting suggestive MRI findings, nonspecific inflammatory tissue, and an early corticosteroid response when disease‐specific investigations and longitudinal data are incomplete. Its clinical relevance is the need to maintain a provisional diagnosis, correlate symptoms with the original imaging and pathology, and reassess competing diagnoses before assigning MS or another chronic demyelinating disorder.

## 2 Case Presentation

### 2.1 Case History

One month before presentation at our institution, a 10‐year‐old boy underwent cranial surgery at another institution after neuroimaging raised concern for a brain tumor. The procedure was recorded as a right frontal craniectomy. The operative report, calendar date, preoperative symptoms and imaging, and perioperative prescriptions were unavailable; therefore, the exact technique and whether the primary purpose was decompression, biopsy, or resection could not be established. Malignancy had not been histopathologically confirmed when he presented to us. No chronic medical illness was reported before this neurosurgical episode.

At the current presentation, he reported progressive right‐hand weakness, finger numbness, and generalized fatigue over several days. Fever, seizures, visual changes, and altered consciousness were not reported. The exact symptom‐onset date and duration were not documented. It was also unclear whether the deficits were new after surgery, persisted from the preoperative period, or recurred after an initial improvement.

Previous neurological episodes, a family history of demyelinating or autoimmune disease, antecedent infection or tuberculosis exposure, and regular or immunosuppressive medication use were not characterized. Vaccination records, Expanded Programme on Immunization (EPI) status, and the names and dates of any recent vaccines were unavailable. Passive smoke exposure, obesity, and vitamin D status were not reported. These gaps prevent assessment of susceptibility factors or temporal associations; they do not support attributing the event to an infection or vaccination.

### 2.2 Neurological Examination

The child was alert and oriented, with a Glasgow Coma Scale score of 15/15. Both pupils reacted to light, and plantar responses were flexor bilaterally. No abnormalities were reported on chest, cardiovascular, abdominal, or genitourinary examination. Weight, vital signs, visual acuity and fields, and funduscopic findings, including the presence or absence of papilledema, were not documented in the examination account. Formal Medical Research Council power grades, grip and fine motor function, tone, deep tendon reflexes, detailed sensory findings, other cranial nerve findings, coordination, gait, and sphincter or meningeal assessments were also unavailable. Unreported findings could not be interpreted as normal.

### 2.3 Investigations

Brain MRI was reported to show two large oval lesions in the right frontoparietal region extending into the centrum semiovale. They were described as centrally hyperintense with a hypointense rim on T2‐weighted sequences and hypointense on T1‐weighted sequences. Peripheral diffusion restriction, incomplete or open‐ring enhancement, perilesional edema, modest mass effect, and a minor midline shift were reported. The spectroscopy interpretation favored demyelination. Scan dates, lesion dimensions, apparent diffusion coefficient (ADC) maps, the measured midline shift, and spectroscopy metabolite ratios were unavailable. Figure 1 reproduces a supplied axial panel; it does not independently establish restricted diffusion or the reported enhancement pattern.

**FIGURE 1:**
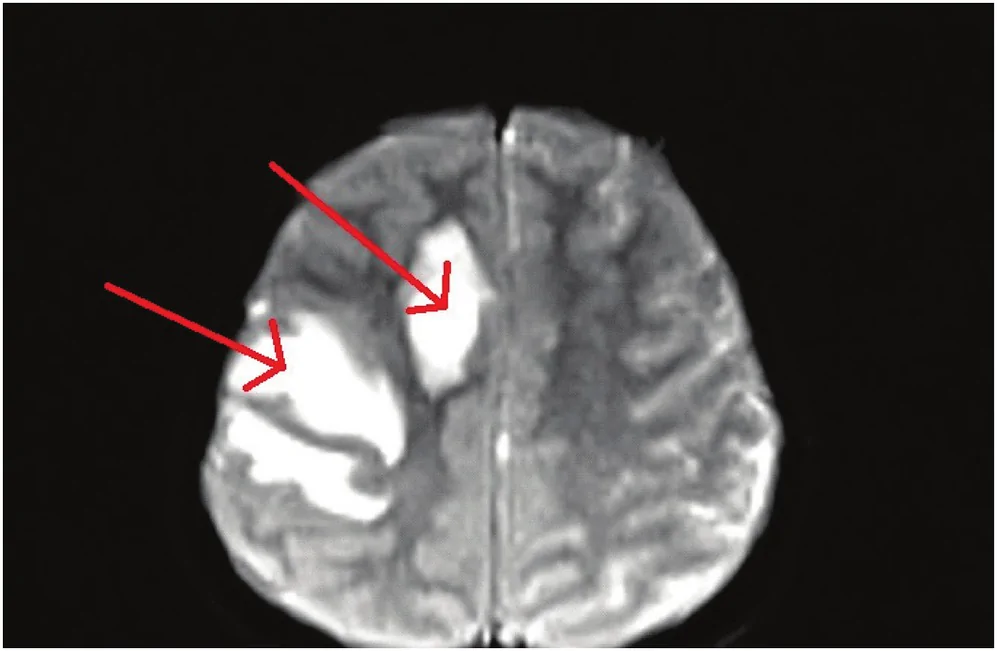
Supplied baseline axial brain MR image showing two areas of high signal in the frontoparietal region (red arrows). The acquisition date, sequence, and anatomical laterality could not be verified from the unlabelled panel. It does not show post‐contrast enhancement; restricted diffusion cannot be confirmed without the corresponding ADC map.

Right‐hand symptoms and right‐sided supratentorial lesions were both recorded. This is a clinical‐radiological laterality discrepancy because a unilateral supratentorial motor lesion would ordinarily produce contralateral weakness. Without the original examination, operative report, and orientation‐labeled imaging, the discrepancy could not be reconciled, and anatomical concordance remains uncertain.

A pathology summary for tissue labeled as the right frontal lesion described several soft‐tissue fragments measuring up to 1 cm. Native glial tissue contained dense sheets of histiocytes, without reported glial neoplasm or atypical mitotic figures. No organisms were described, and special stains were reported as negative, but the stains and microbiological methods were not identified. The specimen date and the procedure yielding the tissue were not specified, so it could not be linked definitively to the earlier operation or a subsequent biopsy. Myelin loss, relative axonal preservation, and results of specific myelin, axonal, or macrophage stains were not documented. The findings supported an inflammatory process but did not specifically confirm demyelination or definitively exclude infection or a sampling‐limited neoplasm.

Cerebrospinal fluid (CSF) was reported as clear, with glucose 68 mg/dL, lactate dehydrogenase below 18 IU/L, fewer than 5 white blood cells/mm^3^, and no red blood cells. Giemsa staining showed occasional lymphocytes. Oligoclonal bands were negative when the result became available at follow‐up. CSF GeneXpert for *Mycobacterium tuberculosis* had been requested, but the final result was unavailable and could not be classified as negative. The collection date, opening pressure, CSF protein, paired serum glucose, and additional culture or infection‐testing results were not available. The indication for lumbar puncture, its timing relative to neuroimaging, and the contemporaneous assessment of contraindications were not documented.

Serum MOG‐IgG and AQP4‐IgG testing was unavailable at the treating center. No spinal MRI report was available, and whether spinal imaging had been performed could not be determined. MOGAD, NMOSD, and additional spinal involvement therefore remained incompletely assessed.

### 2.4 Differential Diagnosis and Diagnostic Assessment

Clinically suspected tumefactive demyelination was the provisional diagnosis, based on the reported MRI pattern, histiocyte‐rich inflammatory tissue, and subsequent clinical and reported radiological improvement. The principal alternatives and the limits of their exclusion are summarized in Table 1. The underlying demyelinating disorder remained unclassified; tumefactive MS was a possibility rather than a confirmed diagnosis.

**TABLE 1:** Differential diagnosis in relation to the present case.

Diagnosis	Findings affecting its likelihood	Remaining uncertainty
Glial neoplasm or CNS lymphoma	No neoplastic elements were reported in sampled tissue; reported interval regression was compatible with an inflammatory process	Sampling limitations remain. Steroid responsiveness does not exclude lymphoma, and the nonspecific histology does not definitively exclude neoplasia
Brain abscess or tuberculoma	Fever was not reported; CSF had fewer than 5 white cells/mm^3^, and no organisms were described in the pathology summary	A focal infection can have bland CSF. Tissue stain methods, relevant cultures, and the final TB GeneXpert result were unavailable; infection was not definitively excluded
Acute disseminated encephalomyelitis	The alert state and absence of reported altered consciousness argue against the encephalopathy required for typical pediatric ADEM [7]	The preceding infection history and complete clinical and MRI pattern were insufficiently characterized
MOG antibody‐associated disease	Childhood onset and large inflammatory lesions are compatible with MOGAD [3, 4]	MOG‐IgG testing was unavailable; MRI response alone cannot distinguish MOGAD from MS‐associated or unclassified demyelination
NMOSD	No visual symptoms were reported, and the available presentation was predominantly cerebral	AQP4‐IgG testing was unavailable and no spinal MRI report was provided. The investigations did not permit adequate assessment of NMOSD

The description of two lesions in one frontoparietal region does not establish involvement of distinct characteristic CNS locations, and regression after treatment does not demonstrate new disease activity over time. Spinal MRI could identify involvement of an additional characteristic location, but spinal imaging alone does not establish dissemination in time. Negative oligoclonal bands do not exclude MS. Nevertheless, the clinical, imaging, and laboratory findings described here were insufficient to establish MS using contemporary diagnostic criteria [6].

### 2.5 Management

The treatment account listed intravenous methylprednisolone 1000 mg once daily and intravenous omeprazole 40 mg. The child's weight, weight‐adjusted steroid dose, infusion duration, administration dates, number of doses, inpatient course, and length of stay were unavailable. Consequently, the appropriateness of the dose on a mg/kg basis and total corticosteroid exposure could not be assessed.

A complete discharge prescription was unavailable. Whether oral prednisone or prednisolone was prescribed, and any starting dose, taper, or stop date could not be established. Use of a steroid‐sparing agent or MS disease‐modifying treatment was also unverified. Blood pressure, glucose, infection surveillance, and other monitoring for corticosteroid toxicity were not described. Adverse events and any treatment given for them were not reported; treatment tolerability could therefore not be determined.

### 2.6 Outcome and Follow‐Up

Hand weakness and finger numbness gradually improved after corticosteroid treatment, with overall symptomatic improvement reported at the one‐month review. Follow‐up MRI was described as showing substantial lesion regression with residual ill‐defined frontoparietal signal abnormality (Figure 2). Follow‐up power grades, detailed sensory findings, grip or fine motor testing, fatigue outcome, daily activities, and school participation were not reported. Dated lesion measurements and matched baseline and follow‐up sequences were unavailable. Improvement was therefore qualitative; neither a percentage recovery nor complete clinical or radiological remission could be established.

**FIGURE 2:**
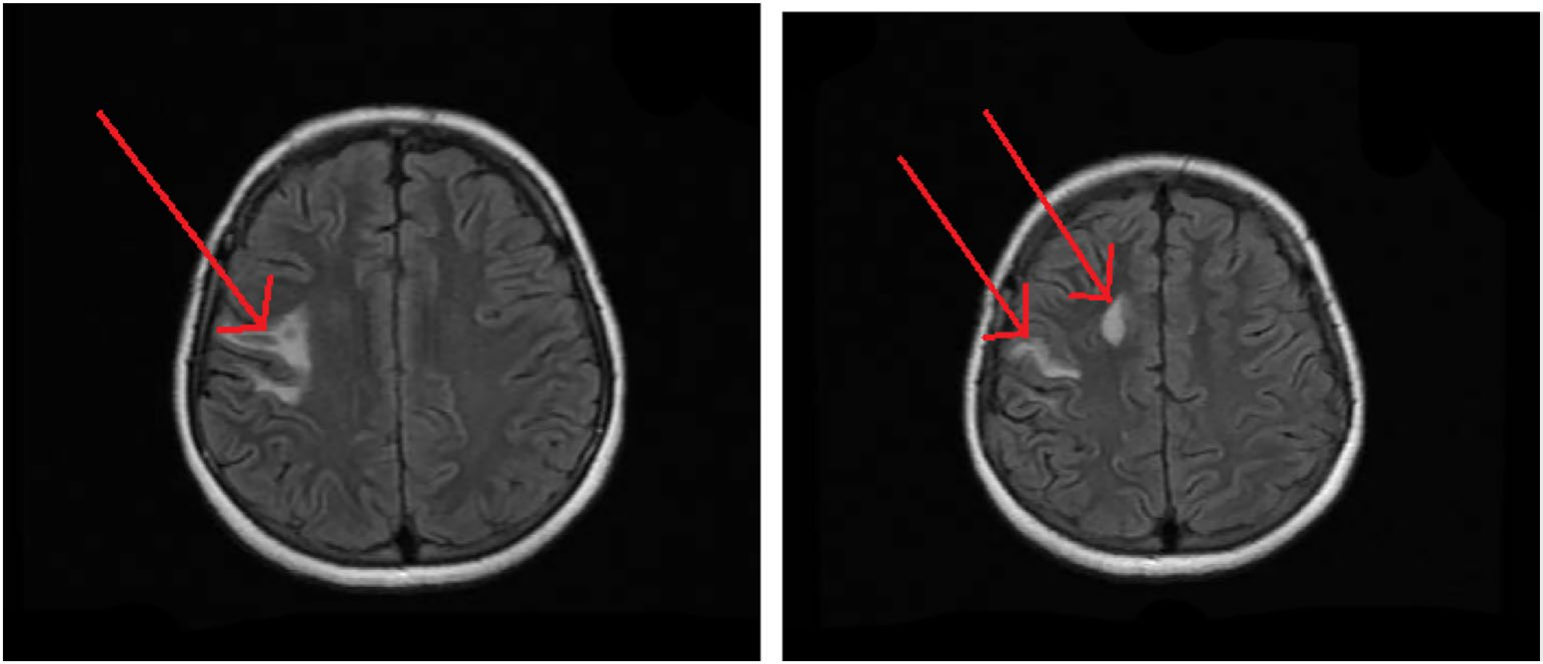
Two supplied axial brain MR images from the reported one‐month follow‐up showing residual frontoparietal signal abnormality (red arrows). Exact scan dates, sequence metadata, and anatomical laterality were unavailable. Regression was described in the follow‐up account, but the displayed baseline and follow‐up panels are unmatched and do not permit a quantitative assessment of lesion regression.

Follow‐up beyond one month was not documented. Subsequent neurology appointment dates, planned brain or spinal MRI, and arrangements for antibody testing were not specified. Referral for physiotherapy, occupational therapy, or hand rehabilitation, and delivery of relapse advice, educational support, or patient and parent counseling were not documented. Recommendations for further assessment and support are discussed below; their implementation in this child could not be established.

## 3 Discussion

This case illustrates the distinction between suspecting a demyelinating lesion pattern and establishing an underlying chronic disease. The MRI narrative, histiocyte‐rich inflammation, and early improvement favored tumefactive demyelination, but none was independently diagnostic. The available evidence did not justify a diagnosis of confirmed MS. The main clinical lesson is that a favorable early response after a presumed tumor operation must be interpreted alongside competing diagnoses and the completeness of the pediatric neuroinflammatory assessment.

MRI features must be weighed together rather than treated as conclusive. Suh et al. identified open or incomplete ring enhancement, a T2‐hypointense rim, and absent or mild mass effect among findings that can favor tumefactive demyelination over primary brain tumors [5]. In this child, the reported enhancement and diffusion pattern supported that interpretation. However, lesion measurements were unavailable, so the usual size threshold of approximately 2 cm could not be independently verified. The supplied panel lacked a matching ADC map and did not display the reported post‐contrast enhancement. Unavailable quantitative spectroscopy and sequence information further limited independent radiological assessment.

Histiocytes without neoplastic elements in the sampled tissue do not establish demyelination. Demonstration of myelin injury with assessment of axonal preservation, interpreted alongside the MRI, would improve diagnostic specificity. Neuropathology review would be particularly relevant if lesions persisted, enlarged, or recurred. Pediatric series show that biopsy can clarify the differential diagnosis and that severe mass effect may require decompressive surgery [2]. Because the original preoperative imaging, operative technique, and surgical indication were unavailable here, the earlier operation cannot be judged unnecessary retrospectively.

Infection remained incompletely excluded. The absence of reported fever and a CSF white‐cell count below 5/mm^3^ do not exclude a localized abscess or tuberculoma. Unspecified negative tissue stains and an unavailable final TB GeneXpert result cannot settle that differential. CSF analysis can contribute to investigation of inflammatory or infectious disease without meningeal symptoms, but this does not establish why lumbar puncture was performed in this child. General procedural guidance requires brain imaging before lumbar puncture when focal deficits or a mass lesion are present and avoidance of puncture when mass effect or pressure gradients create a herniation risk [8]. Without the procedural chronology and assessment of contraindications, the clinical justification and safety of the puncture could not be evaluated retrospectively.

Among inflammatory alternatives, the absence of encephalopathy argues against typical pediatric ADEM [7]. MOGAD remains important because tumefactive lesions occur across antibody‐defined demyelinating disorders and can resemble MS‐associated lesions [3]. The negative oligoclonal bands do not resolve this distinction. Serum MOG‐IgG and AQP4‐IgG testing, appropriate spinal imaging, and follow‐up would be needed to refine the diagnosis; unavailable antibody testing must not be interpreted as a negative result [4].

Other pediatric reports illustrate the same need for diagnostic reassessment. Moro et al. described an atypical pediatric tumefactive presentation [9], and Kamari et al. reported a child with tumefactive MS and Baló‐like lesions [10]. These reports demonstrate the diversity of pediatric tumor‐like demyelinating presentations; they do not establish the underlying diagnosis in the present child.

Pakistani reports provide further comparisons. Ghaouri et al. described a 25‐year‐old man with an earlier episode of transverse myelitis and lesions involving the brain and cervical cord, providing evidence unavailable in this child [11]. Jan et al. reported a 29‐year‐old woman who underwent surgery for suspected glioma, had more specifically characterized macrophage‐rich pathology, and developed new lesions despite treatment [12]. The present child's reported early improvement differed from that refractory course, but uncertain classification and brief follow‐up prevent conclusions about long‐term prognosis.

High‐dose corticosteroids are used for acute inflammatory demyelination, although response is variable. In the pediatric series by Frankl et al., treatment beyond the initial corticosteroid course was frequently required [2]. Plasma exchange or other rescue therapy may be considered for severe refractory disease, while maintenance treatment depends on the established underlying disorder and subsequent course. Pediatric MOGAD recommendations similarly link tapering and maintenance decisions to disease‐specific assessment and relapse history [13]. Such recommendations cannot reconstruct this child's administered regimen or discharge treatment. Missing weight, administration records, and safety observations limit assessment of both treatment exposure and tolerability.

A one‐month response cannot establish a monophasic course or predict freedom from relapse. In the pediatric series by Selek et al., most MS diagnoses were made during follow‐up [1]; recurrent tumefactive disease has also been described in longer‐term cohorts [14]. For children with a similar provisional diagnosis, further assessment should include a documented neurological baseline, interval brain MRI guided by pediatric neurology, and antibody or spinal investigations as clinically indicated. New weakness, sensory loss, visual symptoms, gait change, or bladder symptoms should prompt reassessment. Residual hand impairment may warrant physiotherapy or occupational therapy. Counseling should address diagnostic uncertainty, medication use and adverse effects, relapse recognition, and school participation. These are general recommendations; delivery of these measures was not documented in this child.

### 3.1 Limitations

Diagnostic certainty was limited by unavailable MOG‐IgG and AQP4‐IgG testing, absent spinal imaging results, and nonspecific histology without documented myelin and axonal assessment. Negative CSF oligoclonal bands did not establish an alternative diagnosis. The final TB GeneXpert result and detailed microbiological findings were unavailable, leaving important infectious alternatives incompletely excluded. Original preoperative imaging, lesion measurements, sequence metadata, and the operative report were also unavailable. The symptom‐lesion laterality discrepancy could not be reconciled.

Clinical reporting was further limited by an uncertain symptom‐onset date and relationship to surgery; incomplete neurological, susceptibility‐factor, and vaccination histories; an uncertain lumbar‐puncture chronology; and missing weight, detailed treatment, discharge, toxicity‐monitoring, rehabilitation, and counseling records. Outcomes were qualitative, matched serial imaging was unavailable, and follow‐up extended only to one month. These limitations preclude confirmation of MS, a claim of complete recovery, assessment of treatment safety or long‐term prognosis, or a conclusion that surgery could have been avoided.

## 4 Conclusion

In this 10‐year‐old boy, the reported MRI pattern, histiocyte‐rich inflammatory tissue, and early improvement favored clinically suspected tumefactive demyelination. The underlying disorder remained unclassified, and MS was not confirmed. Interpretation was constrained by incomplete clinical and procedural records, a laterality discrepancy, and short follow‐up. Correlation of the neurological examination with original imaging and neuropathology, completion of feasible disease‐specific investigations, and longitudinal reassessment are necessary to refine the diagnosis.

## Author Contributions

**Fatima Aman:** methodology, project administration, supervision. **Ahmad Ali:** writing – original draft, writing – review and editing, visualization. **Muhammad Fawad Khan:** conceptualization, supervision, investigation. **Haq Nawaz Khan:** conceptualization, investigation, validation. **Asim Shah:** conceptualization, investigation, supervision. **Ali Sher Erkin:** supervision, data curation, resources. **Suleman Khan:** conceptualization, investigation, supervision. **Yasar Sattar:** supervision, resources, conceptualization, investigation. **Aizaz Anwar Khalid:** conceptualization, investigation, supervision.

## Funding

The authors have nothing to report.

## Ethics Statement

This case report was conducted in accordance with the principles of the Declaration of Rehman Medical Institute, Peshawar, Pakistan. Written informed consent was obtained from the patient for publication of this case report and any accompanying images. No institutional ethics committee approval was required for this individual case report; however, patient confidentiality and anonymity were maintained throughout.

## Consent

Written informed consent was obtained from the patient for publication of this case report, including all clinical details and images, in accordance with the journal's policies.

## Conflicts of Interest

The authors declare no conflicts of interest.

## Data Availability

The data that support the findings of this case report are available from the corresponding author upon reasonable request.

## References

[1] A. Selek , R. Göcmen , C. Günbey , B. Konuşkan , I. Oncel , and B. Anlar , “Tumefactive Demyelinating Lesions in Children,” European Journal of Paediatric Neurology 55 (2025): 33–37, 10.1016/j.ejpn.2025.03.005. 40106962

[2] S. Frankl , A. Viaene , A. Vossough , A. Waldman , S. Hopkins , and B. Banwell , “Solitary Tumefactive Demyelinating Lesions in Children: Clinical and Magnetic Resonance Imaging Features, Pathologic Characteristics, and Outcomes,” Pediatric Neurology 157 (2024): 141–150, 10.1016/j.pediatrneurol.2024.05.012. 38917518

[3] L. Cacciaguerra , P. Morris , W. O. Tobin , et al., “Tumefactive Demyelination in MOG Ab‐Associated Disease, Multiple Sclerosis, and AQP‐4‐IgG‐Positive Neuromyelitis Optica Spectrum Disorder,” Neurology 100, no. 13 (2023): e1418–e1432, 10.1212/WNL.0000000000206820. PMC10065219 36690455

[4] B. Banwell , J. L. Bennett , R. Marignier , et al., “Diagnosis of Myelin Oligodendrocyte Glycoprotein Antibody‐Associated Disease: International MOGAD Panel Proposed Criteria,” Lancet Neurology 22, no. 3 (2023): 268–282, 10.1016/S1474-4422(22)00431-8. 36706773

[5] C. H. Suh , H. S. Kim , S. C. Jung , C. G. Choi , and S. J. Kim , “MRI Findings in Tumefactive Demyelinating Lesions: A Systematic Review and Meta‐Analysis,” AJNR. American Journal of Neuroradiology 39, no. 9 (2018): 1643–1649, 10.3174/ajnr.A5775. PMC7655270 30115676

[6] X. Montalban , C. Lebrun‐Frénay , J. Oh , et al., “Diagnosis of Multiple Sclerosis: 2024 Revisions of the McDonald Criteria,” Lancet Neurology 24, no. 10 (2025): 850–865, 10.1016/S1474-4422(25)00270-4. 40975101

[7] L. B. Krupp , M. Tardieu , M. P. Amato , et al., “International Pediatric Multiple Sclerosis Study Group Criteria for Pediatric Multiple Sclerosis and Immune‐Mediated Central Nervous System Demyelinating Disorders: Revisions to the 2007 Definitions,” Multiple Sclerosis 19, no. 10 (2013): 1261–1267, 10.1177/1352458513484547. 23572237

[8] S. Engelborghs , E. Niemantsverdriet , H. Struyfs , et al., “Consensus Guidelines for Lumbar Puncture in Patients With Neurological Diseases,” Alzheimer's & Dementia: Diagnosis, Assessment & Disease Monitoring 8 (2017): 111–126, 10.1016/j.dadm.2017.04.007. PMC5454085 28603768

[9] M. Moro , N. Louhab , and N. Kissani , “Tumefactive Demyelinating Lesions: An Illustrative Pediatric Case With an Atypical Presentation and Literature Review,” Cureus 16, no. 5 (2024): e61207, 10.7759/cureus.61207. PMC11208889 38939300

[10] C. Kamari , E. Galanakis , M. Raissaki , G. Briassoulis , G. Vlachaki , and P. Vorgia , “Pediatric Tumefactive Multiple Sclerosis Case (With Baló‐Like Lesions), Diagnostic and Treatment Challenges,” Neurological Sciences 44, no. 1 (2023): 343–345, 10.1007/s10072-022-06396-y. PMC9816224 36171523

[11] M. S. A. Ghaouri , N. I. Butt , F. Qaisar , M. B. Rasheed , S. Luqman , and S. Ali , “Tumefactive Multiple Sclerosis: A Mimicker of Intracranial Space‐Occupying Lesions,” National Journal of Health Sciences 9, no. 1 (2024): 57–60, 10.21089/njhs.91.0057.

[12] M. M. Jan , I. Ullah , I. Majeed , Z. Ajmal , and M. Tahir , “Tumefactive Demyelinating Lesion Mimicking Glioma: A Diagnostic and Therapeutic Challenge—A Case Report,” SN Comprehensive Clinical Medicine 7 (2025): 159, 10.1007/s42399-025-01912-5.

[13] A. L. Bruijstens , E. M. Wendel , C. Lechner , et al., “E.U. Paediatric MOG Consortium Consensus: Part 5 ‐ Treatment of Paediatric Myelin Oligodendrocyte Glycoprotein Antibody‐Associated Disorders,” European Journal of Paediatric Neurology 29 (2020): 41–53, 10.1016/j.ejpn.2020.10.005. 33176999

[14] I. Pervin , S. Ramanathan , C. Cappelen‐Smith , S. Vucic , S. W. Reddel , and T. A. Hardy , “Clinical and Radiological Characteristics and Outcomes of Patients With Recurrent or Relapsing Tumefactive Demyelination,” Multiple Sclerosis and Related Disorders 82 (2024): 105408, 10.1016/j.msard.2023.105408. 38219394

